# Culture- and PCR-based detection of BV associated microbiological profile of the removed IUDs and correlation with the time period of IUD in place and the presence of the symptoms of genital tract infection

**DOI:** 10.1186/s12941-018-0293-6

**Published:** 2018-11-22

**Authors:** András Ádám, Zoltán Pál, Gabriella Terhes, Márta Szűcs, Israel David Gabay, Edit Urbán

**Affiliations:** 1 Institute of Clinical Microbiology, Albert Szent-Györgyi Clinical Center, Faculty of Medicine, University of Szeged, Semmelweis St. 6, Szeged, 6725 Hungary; 2 Department of Obstetrics and Gynecology, Albert Szent-Györgyi Clinical Center, Faculty of Medicine, University of Szeged, Semmelweis St. 1, Szeged, 6725 Hungary; 3 Department of Obstetrics and Gynecology, Barzilai Medical Center, Ashkelon, Israel

**Keywords:** IUD, Culture and PCR-based detection of bacteria, Biofilm, Upper genital tract infection, PID

## Abstract

**Objectives:**

The long-term use of intrauterine devices (IUDs) may lead to biofilm formation on the surface. The aim of this study was to perform the culture- and PCR-based detection of bacteria/fungi from the biofilm of the removed IUDs with different time periods in place.

**Methods:**

For a 2-year period, 100 IUD users were involved in the study. In the majority of the cases, IUDs were removed because of the patients’ complaints. Beside the aerobic and anaerobic culture, species-specific PCR was carried out to detect *Chlamydia trachomatis Neisseria gonorrhoeae* and the “signalling” bacteria of bacterial vaginosis (BV) in the biofilm removed by vortexing.

**Results:**

Sixty-eight percent of IUDs were used for more than 5 years, 32% were removed after 10 years in place. In 28% of the IUDs ≥ 3 different anaerobic species typically found in BV with or without other aerobic bacteria were found by culture method. *Streptococcus agalactiae* (14%) and *Actinomyces* spp. (18%) were also isolated frequently. The PCR detection of *Gardnerella vaginalis*, *Atopobium vaginae*, *Mobiluncus* spp. and *Ureaplasma urealyticum* were 62%, 32%, 23% and 16%, respectively. Seventy-six percent of the IUDs were PCR positive at least for one “signalling” bacterium of BV. *C. trachomatis* was detected by PCR only in one IUD together with other aerobic and anaerobic bacteria, while the presence of *N. gonorrhoeae* could not be confirmed from the biofilm of these removed devices.

**Conclusion:**

Sexually transmitted infections (STI)-related bacteria—except for one patient—were not detected on the IUDs removed due to different reasons including clinical symptoms of infection. Presence of any BV “signaling” anaerobic bacteria were detected in a much higher number in the biofilm of the removed IUDs by PCR-based method compared to use culture method (76 versus 28 samples). Different aerobic and anaerobic bacteria colonized an equal number of IUDs, independent of the time-period in place, which may be relevant, if the IUD is removed due to planned pregnancy or due to a fear from upper genital tract infection caused by anaerobic bacteria including *Actinomyces* spp.

## Introduction

Nowadays intrauterine devices (IUDs) are accepted as highly effective, long-term methods of contraception. The normal usage period of an IUD is 4–5 years depending on the manufacturer’s recommendation. The use of these devices is usually limited by the concerns about upper-genital-tract infections and forthcoming reproductive health complications [1]. Pelvic inflammatory disease (PID) is the most common gynecological infection, which is associated with high morbidity resulting from inflammation and damage of the reproductive tract. These may lead to severe sequel, such as tubal infertility, ectopic pregnancy and chronic pelvic pain. Many studies have been carried out to determine adequate microbiological aetiology of IUD associated upper genital tract infection [1–3]. The role of classical pathogens of sexually transmitted infections (STI) such as *Chlamydia trachomatis* and *Neisseria gonorrhoeae* in PID was shown not to be higher among women with the use of IUD [4]. However, where STI microbes are not detected before the insertion of the IUD, several authors emphasize the possible role of aerobic and anaerobic bacteria commonly associated with bacterial vaginosis (BV) as the causative agents behind inflammation of the upper genital tract [5, 6]. BV can be described, as a complex alteration of the vaginal flora with the decrease of the hydrogen peroxide-producing lactobacilli replaced by various anaerobic and facultative anaerobic bacteria including some BV-associated bacteria such as *Gardnerella vaginalis*, *Atopobium vaginae*, *Mobiluncus* spp., *Mycoplasma* spp. and *Ureaplasma urealyticum* [7]. IUDs, similarly to other medical implants, as foreign materials may provide a site for biofilm formation and may be a threat for upper genital tract infection [8, 9]. The removal of the IUD, in the case of symptoms of infection, may be advisable [5]. Earlier studies have also reported about different consequences regarding the association of IUD use and BV acquisition: some studies have found a positive correlation, whereas others have not confirmed an increased risk for acquiring BV among IUD users [6, 10]. The possible association between the development of BV and IUD use could be explained by the increased volume and duration of menstrual flow because of the presence of the device [6].

The aim of this study was to detect aerobic/anaerobic bacteria and fungi present in the biofilm cleared away from the surface of the removed IUDs, using classical culture methods and PCR, and to compare these data with the duration of the IUDs in place and the clinical symptoms at the time of the IUD removal.

### Patients and methods

One hundred women using IUDs for different duration of time, were involved in this comparative study. These patients visited the Department of Obstetrics and Gynecology of the University Hospital Szeged, Hungary between January 2014 and December 2015. Beside recording the symptoms and past medical history, all patients had physical examination and pelvic ultrasound prior to the decision of removing the IUD. A written consent was also requested from every participant. Reviewing the number of patients, we set up three groups following the age of the IUD in situ (< 5 years, 5–10 years, > 10 years). As the fewest patient number was among those who wore the device for more than 10 years, we tried to set comparable groups in size up. After the gentle insertion of speculum, IUDs were removed carefully to prevent contamination with the vaginal microflora and were immediately sent to the Institute of Clinical Microbiology.

### Conventional culture methods

All cultures started within 1 h after sampling. Each removed IUD was placed in 10 ml reduced BHI broth (Brain Heart Infusion broth pH 7.2) (Oxoid, Bakingstoke, UK) and mixed on a Vortex shaker for 30 s. After gentle homogenization, half of the suspension was placed in − 80 °C for further investigation by PCR. From the other part of the suspensions, 100 μl was plated on selective (Levine-EMB agar [Conda, Madrid, Spaine], Sabouraud-Chloramphenicol agar, [Bio-Rad, Marnes-la-Coquette, France]) and non-selective (Columbia agar + 5% sheep blood, Chocolate-PolyvitapleX agar [bioMérieux, Lyon, France]) media for isolation of facultative anaerobic bacteria and fungi and Columbia agar supplemented with 5% sheep blood [bioMérieux, Lyon, France] for isolation of strict anaerobic bacteria. For the isolation of anaerobic organisms, plates were incubated in an anaerobic workstation with an atmosphere of 90% N_2_, 5% H_2_, and 5% CO_2_ (Concept 400, Baker-Russkin Co., USA) for 5 days at 37 °C. Mycoplasma IST 2 (bioMérieux, Lyon, France) assay was applied to detect the presence of *Mycoplasma hominis* and *Ureaplasma* spp. after 48 h of incubation using 200 μl inoculum. Selective Thayer-Martin agar (Oxoid, UK) was used to isolate *N. gonorrhoeae* and the plates were incubated for 72 h at 37 °C in 10% CO_2_ atmosphere. Isolated bacteria and fungi were identified by ATB/VITEK (bioMériux, Lyon, France) and/or Microflex LT MALDI-TOF mass spectrometer (Bruker Daltonik, Bremen, Germany).

### Molecular diagnostic methods

Until DNA isolation, the ultrasonicated samples were stored at − 80 °C. DNA was purified using the QIAamp DNA Mini Kit (QIAgen, Hilden, Germany) tissue protocol according to the manufacturer’s instruction. Extracted DNA was subjected to human β-globin real-time PCR using primers PC04 and GH20 to monitor the presence of PCR inhibitors [11]. Traditional species-specific PCR assays were performed using a Veriti 96 Well Thermal Cycler (Applied Biosystems, US) for the detection of *N. gonorrhoeae*, *C. trachomatis*, *U. urealyticum*, *G. vaginalis*, *A. vaginae*, and *Mobiluncus* spp. The sequences of the primers were described earlier [11–16]. Target genes and PCR conditions are shown in Table 1. We used American Type Culture Collection reference strains as positive controls for the PCR assays (Table 1).

**Table 1:** Reference strains, target genes, primer sequences and conditions used for the detection of bacteria with PCR

Species	Target gene	Forward primer (5′–3′)	Amplicon size (bp)	Cycling conditions	References
		Reverse primer (5′–3′)			
*N. gonorrhoeae* ATCC 49981	por A pseudogene	CGGTTTCCGTGCGTTACGA	131	95 °C 10 s, 55 °C 10 s, 72 °C 20 s, 55×	[12]
		CTGGTTTCATCTGATTACTTTCCA			
*C. trachomatis* ATCC VR-902B	MOMP	GGGAAGGTTTCGGTGGAGAT	270	95 °C 40 s, 60 °C 40 s, 72 °C 40 s, 35×	[13]
		AATTACGGTACATTCGTCGCAA			
		CCGTTGAGGGGTTTTCCATTTTTGC			
*U. urealyticum* ATCC 27618	MB antigen	GTATTTGCAATCTTTATATGTTTTCGC	403/448	95 °C 15 s, 60 °C 60 s, 35×	[14]
		CAGCTGATGTAAGTGCAGCATTAAATTC			
*G. vaginalis* ATCC A2508	16S rRNS	GGGCGGGCTAGAGTGCA	207	95 °C 30 s, 62 °C 30 s, 72 °C 30 s, 40×	[15]
		GAACCCGTGGAATGGGCC			
*A. vaginae* ATCC BAA-55	ATOVAGRT3Fw	GGTGAAGCAGTGGAAACACT	276	95 °C 15 s, 62 °C 20 s, 72 °C 40 s, 40×	[11]
		ATTCGCTTCTGCTCGCGCA			
*Mobiluncus* spp. ATCC 35241	16S rRNS	CGCAGAAACACAGGATTGCA	450	94 °C 30 s, 60 °C 30 s, 72 °C 45 s, 40×	[16]
		GTGAACTCCTTTTTCTCGTGAA			

### Statistical evaluation

The comparison of the PCR data was statistically evaluated using the Chi squared test with the Sigmaplot 12.0 program (Systat Sofware, Inc.)

## Results

### Clinical characteristics of patients

The mean age of the patients involved in this study was 44.9 years, ranging between 24 and 73 years. No patient was treated by antibiotics at the time of the removal of the IUD. Due to the bad patient’s compliance towards gynecology services in Hungary, we found some extremely “old” IUDs (nine patients had the IUD in place for ≥ 20 years). The average duration time the IUDs in place was 8.2 years, with a range between 0.5 and 40 years. The patients were put into three groups according to the duration time of the IUD in place: < 5 years (32 patients), 5–< 10 years (36 patients) and ≥ 10 years (32 patients). The mean age of the patients in the three groups at the time of removal of the IUDs are shown in Table 2 together with the clinical symptoms. According to the clinical data at the removal of the IUDs 43% of the patients (equally distributed in the three groups 43.75%, 47.2% and 37.5%, respectively) had no symptoms of infection, but irregular bleeding (in 24 patients), and leiomyoma of the uterus (in 7 patients) were the reasons of removal of the IUD. In all three groups, only few patients visited the gynecologist for the removal of the IUD due to the time in place (Table 2). There were six patients without any symptoms, who wanted to be pregnant and the IUD was removed for this reason and two who became pregnant despite IUD use. Postmenopausal condition was the reason of removal of the IUD in the case of 12 patients. In this cases the insertion of the devices was much earlier, and the patients went through the perimenopausal period with intact IUDs (Table 2). Altogether 57% of the patients had any symptoms of possible infection of the genital tract with or without fever or irregular bleeding, such as acute vaginal/cervical inflammation (n = 30) or severe lower abdominal pain (n = 29) at the time of the removal of the IUD. Postmenopausal conditions of the uterus and cervix could be observed in two patients.

**Table 2:** Clinical symptoms of the patients at the time of removal of the IUD

Clinical symptoms	Number of patients (%)			
	IUD < 5 years	IUD 5–< 10 years	IUD ≥ 10 years	All IUDs
	n 32	n = 36	n = 32	n = 100
Mean age (range)	39.6 (24–51)	43.5 (30–56)	51.8 (33–73)	44.9 (24–73)
No symptoms of infection at the removal of IUD	14 (43.8)	17 (47.2)	12 (37.5)	43 (43)
Removal of IUD due to the time in place	8	11	10	29
Planed pregnancy	3	2	1	6
Pregnancy beside IUD use	1	0	1	2
Irregular bleeding	9	7	8	24
Menopausal/climacteric condition	2	5	5	12
Leiomyoma	1	3	3	7
Any symptoms of infection at the removal of the IUD	18 (56.2)	19 (52.8)	20 (62.5)	57 (57)
Fever ≥ 38 °C	5	7	4	16
Acute vaginal/cervical inflammation	7	8	15	20
Sever lower abdominal pain	5	15	9	29

### Culture results of the removed IUDs

Table 3 shows the culture results of the IUDs according to their different time periods in place. Altogether 62% of the IUDs had positive culture results. Out of the culture positive samples, 30 proved to be positive only for aerobic bacteria with one or two different species (Table 3). The most frequent aerobic bacteria were *Staphylococcus aureus* (10 isolates) *Enterococcus faecalis* (9 isolates) *Streptococcus* spp. excluding *Streptococcus agalactiae* (8 isolates) and *Escherichia coli* (6 isolates). The prevalence of some of these aerobic species are listed separately in the Table 3. *S. agalactiae* colonization of the 14 IUDs was observed, with high colony forming units (CFU), independent how long they were in place. They were also isolated as the only pathogen or together with other aerobic or anaerobic bacteria.

**Table 3:** Culture results of the IUDs removed after different times in place

Culture results	Number of patients (%)			
	IUD < 5 years	IUD 5–< 10 years	IUD ≥ 10 years	All IUDs
	n = 32	n = 36	n = 32	n = 100
Culture negative	11 (34.4)	16 (44.4)	11 (34.4)	38
Culture positive	21 (65.6)	20 (55.6)	21 (65.6)	62
≤ 2 aerobic isolates^a^	11 (34.4)	7 (19.4)	12 (38.4)	30
≥ 3 anaerobic isolates^b^	8 (25.0)	11 (30.5)	9 (28.1)	28
*S. agalactiae* positive^c^	7	4	3	14
*Mycoplasma* positive	0	0	0	0
*U. urealyticum* positive	1	3	2	6
*N. gonorrhoeae* positive	0	0	0	0
*Candida albicans* positive	1	1	0	2

^a^Only aerobic bacteria were isolated alone or together with one other. The following species were found: coagulase-negative *Staphylococcus*, *S. aureus*, Str. *α*-*haemolyticus*, *E. faecalis*, *G. morbillorum*, *Lactobacillus* spp., *E. coli*

^b^Different anaerobic species typically found in BV were isolated (together with other bacteria and fungi): *B. fragilis*, *P. bivia*, *P. intermedia*, *P. melaninogenica*, *P. assacharolytica*, Gram positive anaerobe cocci, *P. assacharolyticus*, *G. vaginalis*, *A. vaginae*, *Mobiluncus* spp., *Actinomyces* spp.

^c^*S. agalactiae* was isolated with high CFU/ml alone or together with some other aerobic or anaerobic bacteria

In the case of 28 IUDs, almost equally distributed in the three groups according to their time in place, a complex anaerobic flora was isolated (≥ 3 anaerobic strains) with or without aerobic species. In the three groups of IUDs, ranged according to their time in place, we had positive culture for ≥ 3 anaerobic bacteria, characteristic for BV, almost in the same percentage (25.0%, 30.5% and 28.1%, respectively). The most frequent anaerobic isolates were Gram-positive anaerobic cocci (GPAC) (24 isolates), *Actinomyces* spp. (18 isolates), *Prevotella* spp. (12 isolates), *Bacteroides* spp. (10 isolates) and *Clostridium* spp. (7 isolates). However, bacteria typically present in BV such as *G. vaginalis*, *A. vaginae*, and *Mobiluncus* spp., were isolated from the samples of the removed IUDs only seldom, such as 1, 2, and 4 cases, respectively (data not shown). Using the MALDI-TOF MS-based identification, out of the 18 *Actinomyces* isolates, 16 were identified on species level as *A. naeslundii* (5), *A. mayeri* (4), *A. odontolyticus* (3), *A. viscosus* (2), and *A. israelii* (2). In the case of two IUDs (removed after 4 and 7 years) only *A. odontolyticus* or *A. naeslundii* were isolated in pure culture after 5 days of anaerobic incubation (data not shown). *Ureaplasma urealyticum* was detected in 6 samples and *M. hominis* and *N. gonorrhoeae* were not detected in any biofilm samples of the IUDs by culture method. *Candida albicans* was isolated only from two IUDs in high CFUs, one that was in place for 3 years and the other for 6.5 years (Table 3).

### Results of PCR detection of bacteria from the ultra-sonicated IUD samples

The specificity of species-specific PCR primer pairs used during this study was successfully confirmed with ATCC reference strains for six bacteria considered to be STI pathogens (*N. gonorrhoeae*, *C. trachomatis*) or frequently found in BV (*G. vaginalis*, *A. vaginae*, *Mobiluncus* spp. and *U. urealyticum*) (Table 1). We have not found any positive PCR reaction for *N. gonorrhoeae* and only one *C. trachomatis* positive result was obtained among the IUDs, which was removed after 17 years in place from a patient who had severe lower abdominal pain. The biofilm of the same IUD was positive for *A. vaginae*, *G. vaginalis* and *U. urealyticum* by PCR and for *A. israelii*, GPACs, *P. oralis* and *E. coli* by the culture method. With the PCR method, 76% of the IUD samples were found to be positive at least for one of the four bacteria frequently found in BV with some increasing percentage according to the time of the IUDs in place (65.6%, 80.6% and 81.3%, respectively) (Table 4). *G. vaginalis* was detected in 62% of the examined IUD samples, *A. vaginae* was present in 32% of the specimens, *Mobiluncus*-specific gene was detected in 23% and *U. urealyticum* PCR was positive for 16% of the samples (Table 4). There was no significant difference in the positive results of any of the BV “signaling” bacteria even not for *A. vaginae* (*p*-0.328) or *Mobiluncus* spp. (*p*-0.192) according to the time of the IUDs in place. Although the numbers of the positive PCR results for these pathogens are increased in the older age IUD groups, no significant differences were found due to the limited number of patients. The combination of the different BV “signaling” bacteria was also evaluated. The most frequently found double positivity was the PCR detection of *G. vaginalis* + *A. vaginae*.

**Table 4:** PCR positivity for *N. gonorrhoeae*, *C. trachomatis* and the BV “signaling” bacteria on IUDs in place for different time periods

Bacteria	Number of positive samples (%)			
	IUD < 5 years	IUD 5–< 10 years	IUD ≥ 10 years	All IUDs
	n = 32	n = 36	n = 32	n = 100
*G. vaginalis*	20 (62.5)	22 (61.1)	20 (62.5)	62
*A. vaginae*	7 (21.8)	13 (36.1)	12 (37.5)	32
*Mobiluncus* spp.	4 (12.5)	9 (25.0)	10 (31.3)	23
*U. urealyticum*	4 (12.5)	8 (22.2)	4 (12.5)	16
*N. gonorrhoeae*	0	0	0	0
*C. trachomatis*	0	0	1 (3.1)	1
Presence of any combination of the 4 BV “signaling” bacteria	9	14	15	38
Presence of any BV “signaling” bacteria	21 (65.6)	29 (80.6)	26 (81.3)	76
*G. vaginalis *+* A. vaginae*	4	7	8	19
*G. vaginalis *+* Mobiluncus* spp.	2	0	3	5
*A. vaginae *+* Mobiluncus* spp.	0	0	0	0
*G. vaginalis *+* A. vaginae *+* Mobiluncus* spp.	2	3	1	6
*G. vaginalis *+* U. uralyticu*m	0	1	1	2
*G vaginalis *+* A. vaginae *+* U. urealyticum*	1	2	2	5
*A. vaginalis *+* Mobiluncus* sp.+* U. urealyticum*	0	0	0	0
*G. vaginalis *+* A. vaginae *+* Mobiluncus* sp. + *U. urealyticum*	0	1	0	1

## Discussion

IUDs are popular contraceptive choices for women however, the possible risk of PID associated with the use of an IUD has been a long-lasting important concern throughout the world. The connection between the development of any upper genital tract infection immediately after the insertion of the IUD, or due to the extended duration of IUD in place has been studied extensively during the past decades, but controversial results were obtained [17]. Although higher rates of PID immediately after insertion of IUDs has been noted in previous studies, some recent data allow for a finer analysis of this relation [2, 18]. Sexually transmitted organisms, especially *N. gonorrhoeae* and *C. trachomatis* were implicated in many cases of PID; however, microorganisms that comprise the vaginal flora (e.g., different anaerobic species dominating the vaginal flora in BV, *G. vaginalis*, *S. aureus*, enteric Gram-negative rods, *Enterococcus* sp. or *S. agalactiae*) have also been associated with PID [19]. In addition, *M. hominis*, *U. urealyticum*, *M. genitalium* and in rare cases, bacteria from the oral region (*Haemophilus influenza* or *Streptococcus pyogenes*) have also been reported to be associated with some cases of PID [20–22]. How the frequency of the use of IUD and the bacteria, which may form a biofilm on it, influences the development of upper genital tract infection, is still a question.

In our study, we used extended incubation time (5 days) for isolation of aerobic and anaerobic bacteria/fungi as well as PCR detection of selected bacteria, which are known to cause female upper genital tract infection such as *N. gonorrhoeae* and *C. trachomatis.* We also detected by PCR some difficult to culture BV “signaling” bacteria, such as *G. vaginalis*, *A. vaginae*, *Mobiluncus* spp. and *U. urealyticum*. Due to the poor patient’s compliance, there were quite a few extremely old IUDs removed with or without symptoms of infection. Only 32% of the patients had an IUD in place for less than 5 years, while the manufacturer’s recommendation is to change the device within 4–5 years, depending on the type. The high number of extremely old IUDs (32% of them were in place for more than 10 years up to 40 years) can probably be explained by the fact that the majority of the examined patient had denied their minor genital complains. No differences were found in culture positivity of the IUDs removed in different time-periods in place. In all three categories of the IUDs, 65.6%, 55.6% and 65.6% of them, respectively, were positive by culture for aerobic and/or anaerobic bacteria (p-0.609) (Table 3). The negative results for *N. gonorrhoeae* using culture- and PCR-based methods, as well as the one positive sample for *C. trachomatis* found by PCR, support the opinion of others that in low risk population IUD usage, even for long time in place, may not increase the possibility of PID caused by STI pathogens [5, 6]. However, it seems that biofilm formation and the presence of different other bacteria (often characteristic for BV) in the biofilm may happen with the same frequency on IUDs in place for different time-periods. Two examples from this study (one from group < 5 years, other from group > 10 years) demonstrates the microbiological flora’s complexity we could detected. An IUD of a 34 year old women, which was inserted only 1.5 year earlier was positive for several bacteria using different method (culture positive for *A. naeslundii*, *Mobiluncus* sp., *Peptostreptococcus anaerobius*, *Prevotella buccalis*, *Prevotella oralis*, *Prevotella loescheii* and PCR positive for *G. vaginalis* and *A. vaginae*). The biofilm removed from another IUD of a 42 years old patient, which was in place for 20 years, was culture positive for *A*. *naeslundii*, *Anaerococcus prevotii*, *Bacteroides fragilis Prevotella bivia*, *Bifidobacterium* sp., *Gemella morbillorum*, *S. agalactiae* and PCR positive for *G. vaginalis* and *A. vaginae* (not shown).

The frequent isolation of *S. agalactiae* from the biofilm of all three groups of IUDs draw the attention that *S. agalactiae* can be a frequent colonizer of the vagina of IUD users, and screening for its presence at the end of the pregnancy is important especially in women who used IUD as birth control earlier [23]. In our study, only two IUDs being in place for 3 and 6.5 years were positive by culture for *C. albicans*. In a recent study, Zahran et al. [24] recovered *Candida* species in 46.4% of 56 IUDs. Non-albicans *Candida* spp. were more frequent among the isolates and strong biofilm formation could be visualized by scanning electron microscope.

The high number of *Actinomyces* isolates found during this study belonging to five different species is in agreement with recent findings about the association of upper-genital-tract infections due to *Actinomyces* spp. in women with IUD in place for different time-periods [23, 25–27]. Clinicians may consider this possibility in case of clinical symptoms of women using IUDs.

Very high number of positive PCR results were observed in all three groups of IUDs detecting the selected bacteria often present in BV, compared with the culture results. Despite the very careful anaerobic culture procedure and the extended incubation time up to 5 days in the anaerobic environment, the confirmation of the possible presence of BV-related bacteria by the culture method in the biofilm on the IUDs was less successful than to detect BV “signaling” bacteria by the PCR method for *G. vaginalis*, *A. vaginae* and *Mobiluncus* spp. It is well known that PCR detection of any bacteria raise the question that non-viable bacteria can also be detected, which may explain this finding. However, the frequent detection of BV associated bacteria or others in the biofilm, present on the surface of the IUDs independent how long time they were in place may draw the attention that in case a severe symptoms culture and PCR-based detection of bacteria and fungi of the removed IUD could help in selection of proper treatment.

## Conclusions

Our study confirms the hypothesis that IUDs, independent of the type or duration of use can increase the possibility of an up-spreading gynecological infection. It is a therapeutic challenge to manage these cases because of the mixed bacterial biofilm flora found on the implant’s surface.

## References

[1] Grimes DA (2000). Intrauterine device and upper-genital-tract infection. Lancet.

[2] Hubacher D (2014). Intrauterine devices & infection: review of the literature. Indian J Med Res.

[3] Taylor BD, Darville T, Haggerty CL (2013). Does bacterial vaginosis cause pelvic inflammatory disease?. Sex Transm Dis.

[4] Birgisson NE, Zhao Q, Secura GM, Madden T, Peipert JF (2015). Positive testing for *Neisseria gonorrhoeae* and *Chlamydia trachomatis* and the risk of pelvic inflammatory disease in IUD users. J Woman’s Health.

[5] Demirezen S, Kucuk A, Beksac MS (2006). The association between copper containing IUCD and bacterial vaginosis. Cent Eur J Public Health.

[6] Madden T, Grentzer JM, Secura GM, Allsworth JE, Peipert JF (2012). Risk of bacterial vaginosis in users of the intrauterine device: a longitudinal study. Sex Transm Dis.

[7] Onderdonk AB, Delaney ML, Fichorova RN (2016). The human microbiome during bacterial vagionsis. Clin Microbiol Rev.

[8] Pal Z, Urban E, Dosa E, Pal A, Nagy E (2005). Biofilm formation on intrauterine devices in relation to duration of use. J Med Microbiol.

[9] Marrie TJ, Costerton JW (1983). A screening and transmission electron microscopic study of the surface of intrauterine contraceptive device. Am J Obstet Gynecol.

[10] Pham AT, Kives S, Merovitz L, Nitsch R, Tessler K, Yudin MH (2012). Screening for bacterial vaginosis at the time of intrauterine contraceptive device insertion: is there a role?. J Obstet Gynaecol Can.

[11] De Backer E, Verhelst R, Verstraelen H, Alqumber MA, Burton JP, Tagg JR (2007). Quantitative determination by real-time PCR of four vaginal *Lactobacillus* species, *Gardnerella vaginalis* and *Atopobium vaginae* indicates an inverse relationship between *L. gasseri* and *L. iners*. BMC Microbiol.

[12] Whiley DM, Buda PJ, Bayliss J, Cover L, Bates J, Sloors TP (2004). A new confirmatory *Neisseria gonorrhoeae* real-time PCR assay targeting the *por*A pseudogene. Eur J Clin Microbiol Infect Dis.

[13] Xia QF, Xu SX, Wang DS, Wen YA, Qin X, Qian SY (2007). Development of a novel quantitative real-time assay using duplex scorpion primer for detection of *Chlamydia trachomatis*. Exp Mol Pathol.

[14] Stellrecht KA, Woron AM, Mishrik NG, Venezia RA (2004). Comparison of multiplex PCR assay with culture for detection of genital mycoplasmas. J Clin Microbiol.

[15] Fredricks DN, Fiedler TL, Thomas KK, Oakles BB, Marrazzo JM (2007). Targeted PCR for detection of vaginal bacteria associated with bacterial vaginosis. J Clin Microbiol.

[16] Schwebke JR, Lawing LF (2001). Prevalence of *Mobiluncus* spp. among women with and without bacterial vaginosis as detected by polymerase chain reaction. Sex Transm Dis.

[17] Hubacher D, Grimes DA, Gemzell-Danielsson K (2013). Pitfalls of research linking the intrauterine device to pelvic inflammatory disease. Obstet Gynecol.

[18] Viberga I, Odlind V, Lazdane G, Kroica J, Berglund L, Olofsson S (2005). Microbiology profile in women with pelvic inflammatory disease in relation to IUD use. Infect Dis Obstet Gynecol.

[19] Ness RB, Kip KE, Hillier SL, Soper DE, Stamm CA, Sweet RL (2005). A cluster analysis of bacterial vaginosis-associated microflora and pelvic inflammatory disease. Am J Epidemiol.

[20] Haggerty CL, Totten PA, Astete SG, Ness RB (2006). *Mycoplasma genitalium* among women with non-gonococcal, non-chlamydial pelvic inflammatory disease. Infect Dis Obstet Gynecol.

[21] Gaydos C, Maldeis NE, Hardick A, Hardick J, Quinn TC (2009). *Mycoplasma genitalium* as a contributor to the multiple etiologies of cervicitis in women attending sexually transmitted disease clinics. Sex Transm Dis.

[22] Pavonen J, Lehtinen M, Teisala K, Heinonen PK, Punnonen R, Aine R (1985). *Haemophylus influenzae* causes purulent salpingitist. Am J Obstet Gynecol.

[23] Lewis R (1998). A review of bacteriological culture of removed intrauterine contraceptive devices. Br J Fam Plan.

[24] Zahran KM, Agban MN, Ahmed SH, Hassen EA, Sabet MA (2015). Patterns of candida biofilm on intrauterine device. J Med Microbiol.

[25] Nakahira ES, Maximiano LF, Lima FR, Ussami EY (2017). Abdominal and pelvic actinomycosis due to longstanding intrauterine device: a slow and devastating infection. Autopsy Case Rep.

[26] Sawtelle AL, Chappell NP, Miller CR (2017). *Actinomyces*-related tubo-ovarian abscess in a poorly controlled type II diabetic with a copper intrauterine device. Mil Med.

[27] Elsayed S, George A, Zhang K (2006). Intrauterine contraceptive device-associated pelvic actinomycosis caused by *Actinomyces urogenitalis*. Anaerobe.

